# Analysis of the influencing factors in the long-term survival of esophageal cancer

**DOI:** 10.3389/fonc.2023.1274014

**Published:** 2024-01-18

**Authors:** Wang Rui, Congcong Li, Qin Da, Yang Yue, Li Jing, Guo Ruirui, Cui Youbin, Tianyu Lu, Bo Li

**Affiliations:** ^1^ School of Public Health, Jilin University, Changchun, China; ^2^ Department of Thoracic Surgery, The First Hospital of Jilin University, Changchun, China

**Keywords:** esophageal squamous cell carcinoma, R0 resection, pathology, blood routine, overall survival

## Abstract

**Background:**

To analyze the prognosis and diagnostic value of relevant hematological indexes on the survival status of patients with esophageal squamous cell carcinoma after radical surgery.

**Methods:**

This study included 206 patients with esophageal cancer who underwent surgical R0 resection. The data, including the basic information, preoperative blood routine, albumin, fibrinogen, surgery-related information, postoperative pathology, and overall survival, of the patients were compared.

**Results:**

The survival and death groups showed a significant difference in overall survival (OS), the degree of differentiation, depth of infiltration, pathological stage, vascular infiltration, nerve infiltration, fibrinogen, white blood cell, neutrophils, platelet, and platelet hematocrit (P<0.05). Tumor located in the middle thorax, larger lesion length, deeper invasion, later pathological stage, vascular infiltration, nerve infiltration, lymph node metastasis, cardiovascular disease, and higher smoking grade were risk factors for poor prognosis of esophageal squamous cell carcinoma (ESCC) (P<0.05). Cardiovascular disease, lower differentiation, tumor located in the middle thorax, and nerve infiltration were independent risk factors for the reduction of survival time of patients with ESCC (P<0.05).

**Conclusions:**

History of cardiovascular disease, tumor located in the middle chest, poorly differentiated esophageal squamous cell carcinoma, visible nerve cancer invasion, hematocrit (HCT), mean erythrocyte hemoglobin concentration (MCHC), and hemoglobin (HB) are independent risk factors for the long-term survival of patients with ESCC.

## Introduction

Esophageal cancer (EC) is a common cancer affecting many regions of the world and carries significant morbidity and mortality (1). The cancer report released in 2021 showed that in 2020, the number of new cases of esophageal cancer worldwide was 604,000, and the number of deaths from esophageal cancer was 544,000 (2). In China, esophageal squamous cell carcinoma (ESCC) is the main type of esophageal cancer and continues to be the sixth leading cause of cancer cases and the fourth leading cause of cancer-related deaths (3). Recently, the clinical strategies for addressing ESCC have focused on a combination therapy involving surgery, chemotherapy, and radiotherapy (4). The choice of treatment is based on the patient’s background and TNM staging according to the American Joint Committee on Cancer (AJCC)/International Union Against Cancer (UICC) clinical stage (5). Currently, there is an emerging immunotherapy method that can be used as an additional treatment. However, tumor development is influenced by various tumor environments and complex heterogeneity. Despite the extensive efforts to combat ESCC and improve the prognosis, it is crucial to identify a reliable biomarker that can provide clinically beneficial information for monitoring the progression of ESCC tumor growth (6).

Nowadays, studies have shown that cancer-related systemic inflammation plays a significant role in the diagnosis and prognosis of cancers, particularly in early stages. Clinicians have access to routine blood test (RBT) data that can provide valuable information. The RBT measures the concentrations of white blood cells, red blood cells, platelets, platelet-to-lymphocyte ratio (PLR), neutrophil-to-lymphocyte ratio (NLR), and monocyte-to-lymphocyte ratio (MLR). These measurements are used to aid in the diagnosis of malignancies, inflammatory diseases, and immune disorders. Furthermore, these indicators are believed to reflect inflammation, nutrition, and/or immunity and have been reportedly associated with the prognosis of patients with esophageal cancer (7, 8).

This study aimed to explore the factors that affect the survival of patients with ESCC by analyzing the post-surgery survival period. Specifically, it aimed to investigate the predictive value of hematological indicators in preoperative examinations on the long-term survival status of patients. The objective is to enable clinicians to formulate more optimized diagnosis and treatment options for patients based on post-surgery risk factors.

## Materials and methods

This study analyzed 206 patients with ESCC who underwent R0 resection in the Department of Thoracic Surgery of the First Hospital of Jilin University from January, 2015 to December, 2020. This study was approved by the Medical Ethics Committee of the First Hospital of Jilin University.

Inclusion criteria were: (1) The pathology of the enrolled patients was squamous cell carcinoma; (2) No neoadjuvant therapy (including chemotherapy, radiotherapy, immunotherapy, and targeted therapy) before surgery; (3) No previous history of tumor; (4) Complete preoperative examination data (gastroscope, computed tomography [CT], upper digestive tract radiography, enhanced CT of chest and abdomen, and color ultrasound of blood vessels of brain and neck); (5) The operation achieved R0 resection (9); and (6) Complete clinical data, including detailed pathological data.

Exclusion criteria were:(1) Subjects who experienced perioperative death or death caused by surgical complications, as well as deaths resulting from factors unrelated to esophageal cancer. (2) Subjects who did not undergo R0 resection (complete tumor removal). (3) Subjects with esophageal non-squamous cell carcinoma and cervical esophageal carcinoma. (4) Subjects who experienced serious complications in other organs during the perioperative period.

### Preoperative data of patients

All patients completed preoperative relevant examinations, including routine blood examination, routine coagulation examination, liver and kidney functions, ions, tumor markers, infection markers and endoscopy, tissue biopsy, upper digestive tract angiography, chest and abdomen enhanced CT, brain CT, intracranial and cervical blood vessels color ultrasound, lower limb veins color ultrasound, electrocardiogram, heart color ultrasound, and pulmonary function examination. Positron-emission CT and coronary CT angiography were selective examinations.

### Operation method

The choice of surgical plan was mainly based on the tumor location. Sweet operation was primarily used for the lower thoracic segment, and the Ivor Lewis and McKeon operations were mainly used for the middle and upper thoracic segments. The specific operation plan was implemented after comprehensive evaluation, according to the actual situation of the patient, such as the location of the lesion, patient’s height, extent of the lesion (tumor length, lymph node metastasis range, etc.), physical function, and patient’s wishes.

### Follow-up

Follow-ups were conducted at 1, 3, and 6 months after operation, with rechecks every 6 months for 2–5 years. After that, it was conducted once a year. We followed up in the form of phone calls and text messages. Follow-up contents: chest and abdomen CT, upper gastrointestinal radiography, cervical lymph node color ultrasound, and tumor markers. The endpoint of follow-up was December 1, 2020 or death. The total survival period was defined as the date of radical surgery to the end of follow-up or death.

### Statistical method

Count data were reported as ratios and composition ratios. Normally distributed continuous variables were reported as mean ± standard deviation, and non-normally distributed continuous variables were reported as M (P25, P75). T-test was used to analyze normally distributed continuous variables between the two groups, Z-test was used to analyze non-normally distributed continuous variables, and the count data were analyzed using two-test or the Fisher’s exact probability method. In univariate analysis, the Kaplan–Meier method was used to plot the survival curve, and log-rank test was used to compare whether the survival curve was statistically different. The Cox regression model was used for multivariate analysis to analyze the impact of various variables on the survival period after esophageal cancer surgery and calculate the hazard ratio (HR) and its 95% confidence interval (CI). All data were analyzed using the Statistical Package for the Social Sciences 24.0 statistical software, and difference of P < 0.05 was considered statistically significant.

## Results

A total of 206 patients with ESCC, who underwent R0 resection, were included. Of the participants, 102 (49.5%) were aged 45 to 59 years, and 104 (50.5%) were aged 60 to 80 years; 196 were men (95.1%), and 10 were women (4.9%); 187 were Han patients (90.8%); 19 patients (9.2%) were from other ethnic minorities. At the end of the follow-up period, the mortality, survival, 3-year overall survival (OS), and 5-year OS rates were 65.0%, 35.0%, 51.0%, and 34.5%, respectively. Detailed information including clinical characters is shown in **Table 1**.

**Table 1:** Baseline information of all the participants.

Characters		All (N=206)
Age (y)		60.21 ± 8.19
BMI (kg/m^2^)		22.31 ± 2.82
Sex	Male	196 (95.1%)
	Female	10 (4.9%)
Nationality	Han	187 (90.7%)
	Mongolian	17 (8.3%)
	Others	2 (1.0%)
Hypertension	No	176 (85.4%)
	Yes	30 (14.6%)
Diabetes	No	191 (92.7%)
	Yes	15 (7.3%)
Cardiovascular disease	No	199 (96.6%)
	Yes	7 (3.4%)
Smoke	No	47 (22.8%)
	Yes	159 (77.2%)
Drink	No	30 (14.6%)
	Yes	176 (85.4%)
Smoking classification (10)	Light	85 (41.3%)
	Moderate	70 (34.0%)
	Severe	51 (24.7%)
Alcohol intake classification (11)	Low	36 (17.5%)
	Moderate	19 (9.2%)
	High	25 (12.1%)
	Very High	126 (61.2%)
Operation mode	Sweet	73 (35.4%)
	Ivor-Lewis	93 (45.1%)
	McKeown	40 (19.5%)
Anesthesia classification	I	20 (9.7%)
	II	124 (60.2%)
	III	60 (29.1%)
	IV	2 (1.0%)
Degree of tumor differentiation	Low	26 (12.6%)
	Medium-low	44 (21.4%)
	Medium	124 (60.2%)
	High-medium	8 (3.9%)
	High	4 (1.9%)
Number of lesions		1.06 ± 0.25
Length of lesion	<3cm	85 (41.3%)
	3-5cm	71 (34.5%)
	>5cm	50 (24.3%)
Tumor location	Upper thoracic segment	11 (5.3%)
	Middle thoracic segment	46 (22.3%)
	Inferior thoracic segment	149 (72.3%)
Degree of infiltration	Mucosal layer	6 (2.9%)
	Submucosa	38 (18.4%)
	Muscularis propria	31 (16.0%)
	Adventitia	129 (62.6%)
Pathological staging	IB	28 (13.6%)
	IIA	17 (8.3%)
	IIB	44 (21.4%)
	IIIA	21 (10.2%)
	IIIB	96 (46.6%)
Lifetime (months)		42.98 ± 30.65
Number of lymph node dissection		17.39 ± 9.82
Lymph node clearance	<15	76 (36.9%)
	≥15	130 (63.1%)
Number of positive lymph nodes		1.50 ± 1.95
Lymph node metastasis	No	82 (39.9%)
	Yes	124 (60.2%)
LNR (%)		9.86 ± 13.67
LODDS		-1.01 ± 0.47
vascular invasion	No	101 (49.0%)
	Yes	105 (51.0%)
Nerve infiltration	No	127 (61.7%)
	Yes	79 (38.3%)
Postoperative adjuvant therapy	No	71 (34.5%)
	Yes	89 (43.2%)
	Unclear	46 (22.3%)
Fibrinogen (FIB) (g/L)		3.24 ± 0.84
Albumin (ALB) (g/L)		39.20 ± 3.85
White blood cell (WBC) (×10^9^/L)		6.42 ± 1.89
Neutrophils (NE) (×10^9^/L)		3.94 ± 1.61
Lymphocyte (LY) (×10^9^/L)		1.89 ± 0.65
Monocyte (MO) (×10^9^/L)		0.45 ± 0.20
Eosinophils (EO) (×10^9^/L)		0.11 (0.07-0.21)
Basophil (BA) (×10^9^/L)		0.01 (0.01-0.03)
Red blood cell (RBC) (×10^12^/L)		4.55 ± 0.50
Hemoglobin (HB) (g/L)		142.81 ± 14.47
Hematocrit (HCT) (%)		0.43 ± 0.04
Mean red blood cell volume (MCV) (fL)		94.30 ± 5.00
Mean erythrocyte hemoglobin (MCH) ( pg)		31.46 ± 1.78
Mean erythrocyte hemoglobin concentration (MCHC) (g/L)		333.72 ± 10.08
Erythrocyte distribution width (RDW) (%)		13.04 ± 0.82
Platelet (PLT) (×10^9^/L)		228.05 ± 63.90
Platelet hematocrit (PCT) (%)		0.24 ± 0.06
Mean platelet volume (MPV) (fL)		10.79 ± 0.86
Platelet distribution width (PDW) (%)		12.98 ± 7.18
Neutrophil-to-lymphocyte ratio (NLR)		2.45 ± 2.70
Platelet-to-lymphocyte ratio (PLR)		134.30 ± 66.06
Monocyte-to-lymphocyte ratio (MLR)		0.26 ± 0.14
Albumin-to-fibrinogen ratio (AFR)		12.86 ± 3.82

**Figure 1:**
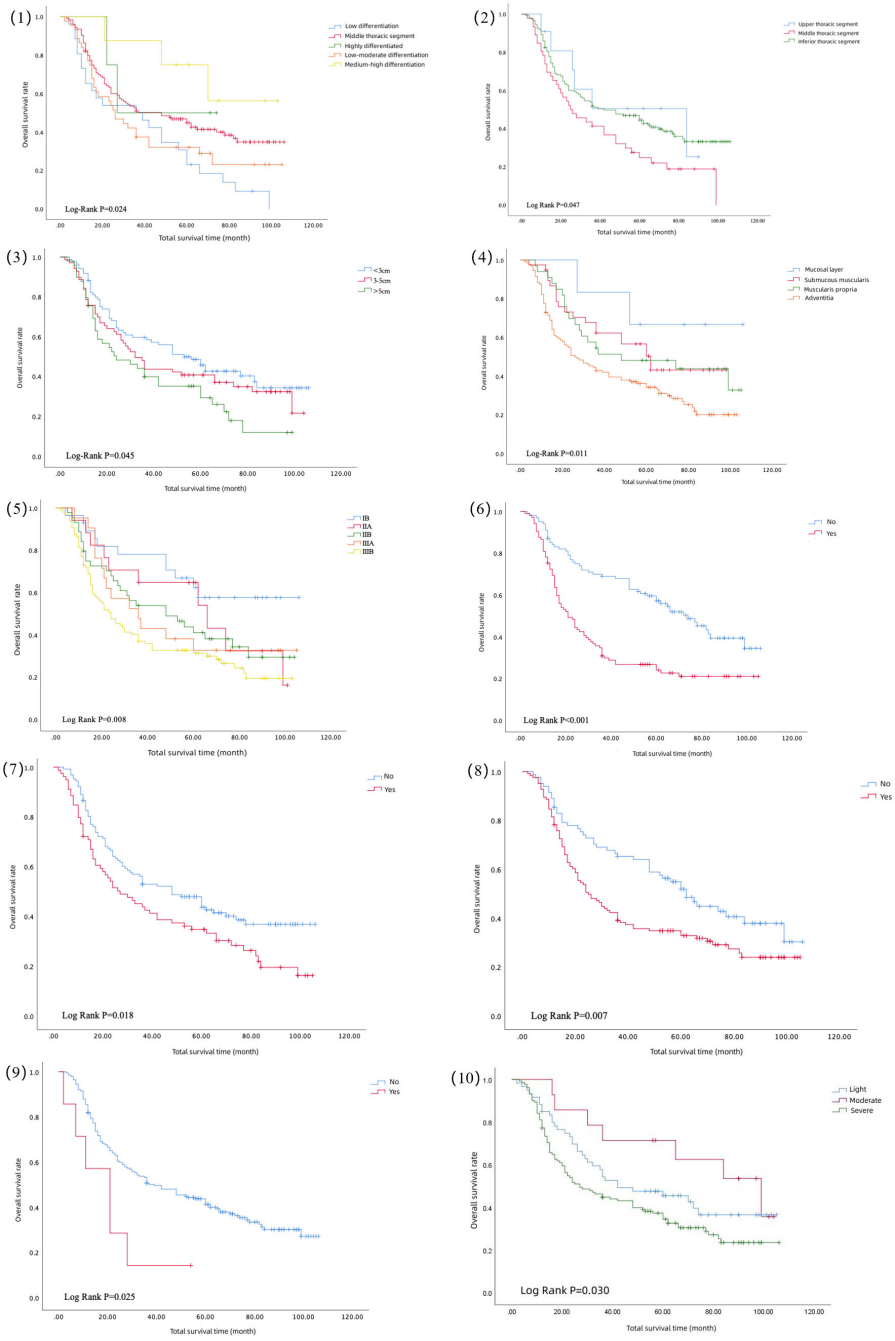
Survival curve of differentiation degree (1), tumor location (2), tumor size (3), invasion degree (4), pathological stage (5), vascular invasion (6), nerve invasion (7), lymph node metastasis (8), preoperative cardiovascular disease (9), and smoking level (10).

The results of the univariate analysis are shown in **Table 2**, which shows that the higher the degree of differentiation, the longer the survival period after surgery. The survival curves of moderately and well-differentiated ESCC were significantly higher. As shown in **Figure 1**(1), the prognoses of moderately and highly differentiated ESCC were better than that of poorly and moderately poorly differentiated ESCC. As shown in **Figure 1**(2), the long-term survival of patients with ESCC in the middle thoracic segment was significantly lower than that in the upper and lower thoracic segments. The longer lesion length, shown in **Figure 1**(3), indicated poor long-term survival after surgery. The survival curves of ESCC with different invasion depths were significantly different, as shown in **Figure 1**(4), suggesting that the tumor breaking through the submucosa significantly reduced the survival period. In **Figure 1**(5), the survival curve showed that the survival period decreased significantly with the upgrading of pathological stages. The survival curve of patients without vascular invasion was significantly higher than that of patients with vascular invasion, as shown in **Figure 1**(6). Postoperative pathology showed that the median survival period of patients with tumor infiltrating peripheral nerves was lower than that of patients without nerve infiltration, as shown in **Figure 1**(7). The survival curve of patients with lymph node metastasis was significantly reduced, indicating poor prognosis, as shown in **Figure 1** (8). In addition, this study also found that preoperative cardiovascular disease is an important risk factor for poor prognosis after ESCC. The median survival period of patients without cardiovascular disease was 39 months, and the median survival period of patients with cardiovascular disease was 21 months. There was a significant difference between the two survival curves, as shown in **Figure 1**(9). And **Figure 1**(10) shows that the survival time of patients with severe smoking was shorter.

**Table 2:** The Kaplan Meier survival analysis results.

Characters		Median survival time (months)	Survival time 95%CI		χ^2^	P
			Lower	Upper		
Differentiation degree	Low differentiation	36	4.77	67.23	11.234	0.024
	Low-medium differentiation	26	23.23	72.77		
	Mesodifferentiation	48	17.07	34.93		
	Medium-high differentiation	49	58.26	101.11		
	Highly differentiated	27	24.93	73.57		
Tumor location	Upper thoracic	84	22.29	145.71	6.110	0.047
	Middle thoracic	25	11.71	38.29		
	Inferior thoracic	42	21.55	62.46		
Length of lesion	<3cm	53	36.87	69.13	6.102	0.049
	3-5cm	32	23.90	40.10		
	>5cm	24	7.16	40.84		
Degree of infiltration (N,%)	Mucosal layer	62	44.49	79.51	11.170	0.011
	Submucosa	48	0.00	100.14		
	Muscularis propria	26	15.82	36.19		
	Adventitia	36	24.15	47.85		
Pathological staging	IB	75	61.09	90.03	13.800	0.008
	IIA	66	26.05	105.95		
	IIB	48	19.01	77.00		
	IIIA	36	16.56	55.44		
	IIIB	24	16.23	31.77		
Vascular invasion	No	74	57.47	90.53	22.154	<0.001
	Yes	21	15.19	26.81		
Nerve infiltration	No	48	30.29	65.71	5.605	0.018
	Yes	27	13.62	40.38		
Lymph node metastasis	No	62	49.12	74.88	7.190	0.007
	Yes	25	17.50	32.51		
Cardiovascular disease	No	39	24.88	53.12	5.030	0.025
	Yes	21	9.29	32.71		
Smoking level	Light	53	36.87	69.13	6.219	0.045
	Moderate	32	23.90	40.10		
	Severe	24	7.16	40.84		

The Cox multifactorial results are shown in **Table 3**. Cardiovascular disease was an independent risk factor for the long-term prognosis of ESCC after surgery (P = 0.006), and the probability of death was 5.776 times higher than that without cardiovascular disease (HR = 5.776). The death risk of poorly differentiated ESCC was approximately 2.5 times higher than that of moderately differentiated ESCC, and the degree of differentiation was an independent risk factor for long-term postoperative ESCC (P = 0.004). The death risk of ESCC in the middle segment was 4.234 times higher than that in the upper segment (HR = 4.234), and tumor location was an independent risk factor (P = 0.015). The death risk of tumor tissue infiltrating peripheral nerves increased 2.394 times (HR = 2.394). Nerve infiltration was an independent risk factor for the long-term prognosis of ESCC after surgery. Preoperative HCT (P=0.037) and MCHC (P=0.040) were independent risk factors for the long-term survival of ESCC.

**Table 3:** The Cox multifactor analysis.

Characters		HR	95% CI		*P*
			Lower	Upper	
Cardiovascular disease	Yes vs No	5.776	1.643	20.238	0.006
Differentiation degree	Medium-low vs low	0.401	0.216	0.745	0.004
Medium vs low	0.822	0.156	4.335	0.817
	High-medium vs low	0.434	0.214	0.880	0.021
	High vs low	0.156	0.040	0.607	0.007
Tumor location	Middle vs Upper	4.234	1.327	13.507	0.015
	Inferior vs Upper	2.166	0.731	6.420	0.163
Nerve infiltration	Yes vs No	2.394	1.386	4.137	0.002
HB		0.621	0.402	0.958	0.031
HCT		1.756	3.818	8.678	0.037
MCHC		1.475	1.010	2.137	0.040

*Cox regression included gender, age, BMI, nationality, diabetes history, hypertension history, smoking history and drinking history to balance the influence of various factors.

## Discussion

In this study, the 3-year OS of patients with esophageal cancer who received R0 resection was 51.0%, and the 5-year OS was 34.5%, which was much higher than the overall 5-year OS of esophageal cancer (~10%), consistent with the 5-year OS (~15% - 40%) after esophagectomy (12). There are few reports about the influence of circulatory system diseases on the prognosis of esophageal cancer, and there are individual reports that hypertension is a risk factor for the prognosis of esophageal cancer (13). This study explores the influence of preoperative cardiovascular disease on the prognosis. The results of single factor Kaplan–Meier analysis show that preoperative cardiovascular disease has a bad effect on postoperative survival, and the survival curve can directly show the difference between the two. The Cox multivariate analysis showed that the death rate of patients with preoperative cardiovascular disease was 5.776 times higher than that of patients without preoperative cardiovascular disease. It can be seen that preoperative cardiovascular disease is an independent risk factor for poor prognosis of ESCC. The probable cause behind this association is that esophageal cancer patients with cardiovascular disease tend to experience higher postoperative complications. Moreover, during the recovery period after surgery, most esophageal cancer patients commonly face weight loss and a decline in their nutritional status. Patients with pre-existing cardiovascular disease generally exhibit low tolerance towards these conditions, which ultimately results in an unfavorable postoperative prognosis. This is an important finding of this study, suggesting that ESCC patients with cardiovascular disease should prioritize long-term monitoring and control of their condition.

In addition, this study’s results support that TNM staging is a risk factor for poor prognosis of patients with ESCC after surgery. The previous literature believed that even patients with the same TNM stage had different postoperative survival periods (10). Some scholars found that factors, such as lesion length, nerve invasion, and vascular invasion, are independent risk factors of esophageal cancer prognosis, which should also be attributed to tumor invasion and become evaluation indicators when predicting the esophageal cancer survival period (11, 14–16). At present, the depth of invasion is the basis for evaluating the T stage in the TNM stage. Studies have proved that the depth of invasion is an independent risk factor for the prognosis of patients with ESCC with negative lymph nodes. The survival period decreases with the increase in the depth of invasion (17). This study’s results show that the depth of invasion affects the survival of patients with ESCC after surgery, further supporting this result.

In recent years, more and more experts have realized the value of vessel or nerve infiltration for prognosis and survival, and related research has become increasingly fierce (18–21). This study also reached similar conclusions. Therefore, this study believes that postoperative pathology suggests that vascular or nerve invasion should be further treated with postoperative adjuvant therapy to prolong the total survival period. Currently, there is controversy surrounding the relationship between tumor location and the prognosis of esophageal cancer. Some research suggests that patients with tumors located in the upper and middle regions have a better surgical prognosis compared to those with tumors in the lower region (22). However, other studies (23) indicates that the survival rate of upper thoracic esophageal cancer was lower than that of middle and lower esophageal cancer. This is because upper thoracic esophageal cancer is generally believed to be in close proximity to the larynx and recurrent laryngeal nerve. It is difficult to operate on and can easily result in anastomotic leakage after surgery. There are also some additional studies have found that the difference in tumor location does not have a statistically significant impact on patient prognosis (24, 25). The findings of this study are consistent with Chen’s research (26), suggesting that the median survival time of patients with mid-thoracic ESCC is significantly lower than that of the patients with ESCC in the upper and lower segments. This can be attributed to the fact that the middle segment of the esophagus is deeper, surrounded by vital organs and structures, and has comparably less blood supply than the other segments.

Many markers routinely obtained from peripheral blood have proved to be predictors for the prognosis of different types of cancer (27–29). It is believed that tumor-related inflammatory reactions can interfere with the prognosis and final outcome of esophageal cancer patients by promoting angiogenesis, distant dissemination, interfering with immune response, and influencing antitumor treatment (30). A study on patients with ESCC found that the prognosis of patients with high-level HB before operation was better than that of patients with low-level HB, and the best cut-off value was 132.5 g/L (31). However, a nationwide retrospective study in Finland on patients with esophageal cancer showed that preoperative HB levels were not related to the prognosis (32). This study found that high HB had a protective effect on long-term prognosis, possibly due to high HB indicating a good preoperative nutritional status. Previous studies have shown that preoperative nutritional risk screening has predictive value for the prognosis of patients with esophageal cancer, and prognostic nutritional indicators can objectively reflect the nutritional status of patients by calculating hematological parameters, thus indicating the long-term survival of patients (33, 34). In addition, this study also found low HCT had a statistically significant impact on survival, which is consistent with the research findings of B Mungo et al. (35). They believe that low HCT and MCHC are independent risk factors for poor prognosis in esophageal cancer patients. This may be attributed to the patients’ low red blood cell count and low hemoglobin levels prior to surgery, which elevates the probability of developing postoperative anemia. The aforementioned indicators suggest that doctors can enhance their focus on the preoperative nutritional status of patients with esophageal cancer by closely monitoring serological indicators that reflect nutritional status (36). This approach may contribute to improving the patients’ postoperative survival rates. Some other blood markers that were previously identified to influence tumor prognosis did not exhibit specific effects in this study. Therefore, large-scale cohort studies are still needed to verify the relationship between routine blood examination and ESCC prognosis.

Limitations of this study were (1) This study only included patients with ESCC in this center, lacking a multicenter data support. (2) The sample size of this study was small, and the results need to be further validated for research validation. (3) During the follow-up of this study, we asked all patients about their disease-free and disease-specific survival; however, many patients did not have regular reexaminations, including many who refused to accept the examination due to economic reasons and their abandonment of treatment after recurrence, which was another limitation of this study. Larger sample size and multicenter studies are necessary.

## Conclusions

History of cardiovascular disease, tumor located in the middle chest, poorly differentiated esophageal squamous cell carcinoma, visible nerve cancer invasion, high HCT, high MCHC, and low HB are independent risk factors for the long-term survival of patients with ESCC.

## Data availability statement

The original contributions presented in the study are included in the article/**Supplementary Material**. Further inquiries can be directed to the corresponding authors.

## Ethics statement

The studies involving humans were approved by the Medical Ethics Committee of the First Hospital of Jilin University. The studies were conducted in accordance with the local legislation and institutional requirements. The participants provided their written informed consent to participate in this study.

## Author contributions

WR: Conceptualization, Investigation, Methodology, Software, Writing – original draft, Writing – review & editing. CL: Data curation, Investigation, Writing – review & editing. QD: Data curation, Investigation, Writing – review & editing. YY: Data curation, Methodology, Writing – review & editing. LJ: Investigation, Software, Writing – review & editing. GR: Methodology, Software, Writing – review & editing. CY: Conceptualization, Resources, Writing – review & editing. TL: Conceptualization, Funding acquisition, Resources, Writing – review & editing. BL: Conceptualization, Investigation, Resources, Software, Writing – review & editing.
