# Supplementary figures and images for: Analysis of the influencing factors in the long-term survival of esophageal cancer
Source: Front Oncol. 2024 Jan 18;13:1274014. doi: 10.3389/fonc.2023.1274014 (PMC10833228; doi:10.3389/fonc.2023.1274014)

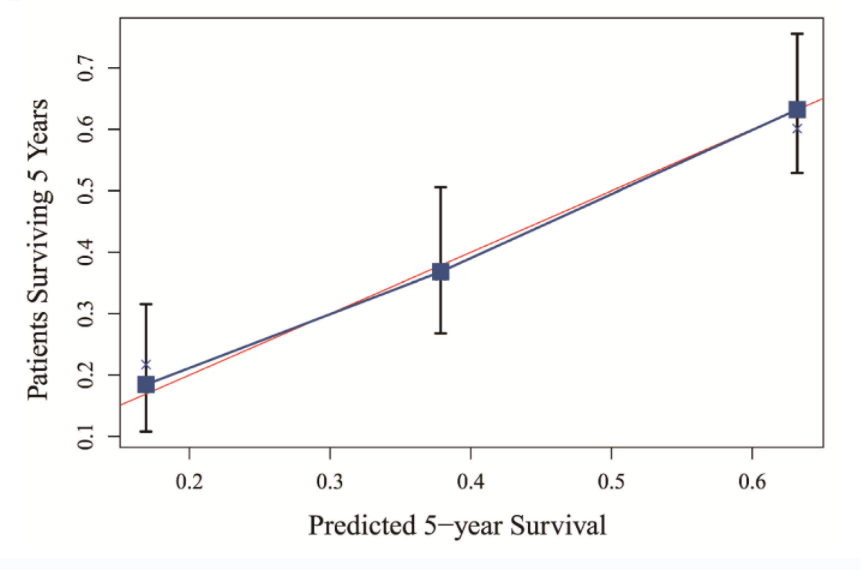

Supplement: Supplementary file 1 [file Image_1.jpeg]
